# A multicenter study confirms *CD226 *gene association with systemic sclerosis-related pulmonary fibrosis

**DOI:** 10.1186/ar3809

**Published:** 2012-04-24

**Authors:** Lara Bossini-Castillo, Carmen P Simeon, Lorenzo Beretta, Jasper C Broen, Madelon C Vonk, Raquel Ríos-Fernández, Gerard Espinosa, Patricia Carreira, María T Camps, Maria J Castillo, Miguel A González-Gay, Emma Beltrán, María del Carmen Freire, Javier Narváez, Carlos Tolosa, Torsten Witte, Alexander Kreuter, Annemie J Schuerwegh, Anna-Maria Hoffmann-Vold, Roger Hesselstrand, Claudio Lunardi, Jacob M van Laar, Meng May Chee, Ariane Herrick, Bobby PC Koeleman, Christopher P Denton, Carmen Fonseca, Timothy RDJ Radstake, Javier Martin

**Affiliations:** 1 Instituto de Parasitología y Biomedicina López-Neyra, IPBLN-CSIC, Avenida del Conocimiento s/n, Granada, 18100, Spain; 2 Servicio de Medicina Interna, Hospital Valle de Hebron, Passeig de la Vall d'Hebron, 119-129, Barcelona, 08035, Spain; 3 IRCCS Fondazione Policlinico-Mangiagalli-Regina Elena and University of Milan, Allergy, Clinical Immunology and Rheumatology, Via Francesco Sforza 28, Milan, 20122, Italy; 4 Department of Rheumatology, Radboud University Nijmegen Medical Centre, Geert Grooteplein Zuid 10, Nijmegen, 6525 GA, The Netherlands; 5 Servicio de Medicina Interna, Hospital Clínico Universitario, Avenida Doctor Olóriz 16, Granada, 18012, Spain; 6 Servicio de Enfermedades Autoinmunes, Hospital Clinic, C/Villarroel, 170, Barcelona, 08036, Spain; 7 Servicio de Reumatología, Hospital 12 de Octubre, Avenida de Córdoba, s/n, Madrid, 28041, Spain; 8 Servicio de Medicina Interna, Hospital Carlos Haya, Avenida Carlos Haya s/n, Málaga, 29010, Spain; 9 Servicio de Medicina Interna, Hospital Virgen del Rocío, Avenida Manuel Siurot s/n, Sevilla, 41013, Spain; 10 Servicio de Reumatología, Hospital Universitario Marqués de Valdecilla, IFIMAV, Avenida Valdecilla 25, Santander, 39008, Spain; 11 Servicio de Reumatología, Hospital del Doctor Peset Aleixandre, Avenida Gaspar Aguilar 90, Valencia, 46017, Spain; 12 Unidad Trombosis y Vasculitis, Servicio de Medicina Interna, Hospital Xeral-Complexo Hospitalario Universitario de Vigo, Rua Pizarro 22, Vigo, 36204, Spain; 13 Servicio de Reumatología, Hospital Universitario de Bellvitge, C/Feixa Llarga s/n, 08907, Barcelona, Spain; 14 Servicio de Medicina Interna, Hospital Parc Taulí, Parc del Taulí s/n, Sabadell, 08208, Spain; 15 Hannover Medical School, Carl-Neuberg-Straße 1, Hannover, 30625, Germany; 16 Ruhr University of Bochum, Universitätsstraße 150, Bochum, 44801, Germany; 17 Department of Rheumatology, Leiden University Medical Center, Albinusdreef 2, Leiden, 2333 ZA, The Netherlands; 18 Department of Rheumatology, Rikshospitalet, Oslo University Hospital, Rikshospitalet-Radiumhospitalet Medical Center, Oslo, 0027, Norway; 19 Department of Rheumatology, Lund University, Paradisgatan 2, Lund, SE-221 00, Sweden; 20 Department of Medicine, Università degli Studi di Verona, Via dell'Artigliere, 19, Verona 37129, Italy; 21 Institute of Cellular Medicine, Newcastle University, Framlington Place, Newcastle upon Tyne, Newcastle, NE2 4HH, UK; 22 Centre for Rheumatic Diseases, Glasgow Royal Infirmary, 84 Castle Street, Glasgow G4 0SF, UK; 23 Arthritis Research UK Epidemiology Unit, The University of Manchester, Manchester Academic Health Science Centre, Stopford Building, Oxford Road, Manchester, M13 9PT, UK; 24 Section Complex Genetics, Department of Medical Genetics, University Medical Center Utrecht, Universiteitsweg 100, Utrecht, 3584 CG, The Netherlands; 25 Centre for Rheumatology, Royal Free and University College Medical School, University College London, Royal Free Campus, Rowland Hill Street, London, NW3 PF, UK; 26 Department of Rheumatology and Clinical Immunology, University Medical Center Utrecht, Heidelberglaan 100, Utrecht, 3584 CX, The Netherlands

## Abstract

**Introduction:**

*CD226 *genetic variants have been associated with a number of autoimmune diseases and recently with systemic sclerosis (SSc). The aim of this study was to test the influence of *CD226 *loci in SSc susceptibility, clinical phenotypes and autoantibody status in a large multicenter European population.

**Methods:**

A total of seven European populations of Caucasian ancestry were included, comprising 2,131 patients with SSc and 3,966 healthy controls. Three *CD226 *single nucleotide polymorphisms (SNPs), rs763361, rs3479968 and rs727088, were genotyped using Taqman 5'allelic discrimination assays.

**Results:**

Pooled analyses showed no evidence of association of the three SNPs, neither with the global disease nor with the analyzed subphenotypes. However, haplotype block analysis revealed a significant association for the TCG haplotype (SNP order: rs763361, rs34794968, rs727088) with lung fibrosis positive patients (*P*_Bonf _= 3.18E-02 OR 1.27 (1.05 to 1.54)).

**Conclusion:**

Our data suggest that the tested genetic variants do not individually influence SSc susceptibility but a *CD226 *three-variant haplotype is related with genetic predisposition to SSc-related pulmonary fibrosis.

## Introduction

Systemic sclerosis (SSc) is a connective tissue disorder in which fibrotic collagen deposition, vascular damage, autoimmunity and autoantibody production (especially anticentromere (ACA), and antitopoisomerase, (ATA) antibodies) are the main hallmarks [1]. SSc patients can be classified classically in two major subgroups, those suffering from limited cutaneous SSc (lcSSc) and those with the diffuse cutaneous form of the disease (dcSSc) [2].

The genetic component of SSc has been recently reinforced and several genes involved in immune regulation have been proposed as risk factors for the development of SSc [3]. A number of loci such as *IRF5 *[4], *STAT4 *[5,6], *BANK1 *[7,8], *C8orf13-BLK *[9,10], *CD247 *[11,12] and *TNFSF4 *[13,14], have been associated with genetic predisposition to SSc in Caucasian populations. Some of these loci are shared with other autoimmune diseases, such as rheumatoid arthritis (RA) and systemic lupus erythematosus (SLE), reinforcing the theory of a common genetic background in autoimmune diseases [15].

CD226 (cluster of differentiation 226)/PTA1 (platelet and T-cell activation antigen 1)/DNAM-1 (DNAX accessory molecule 1) is a member of the immunoglobulin superfamily and it plays an important role in the co-stimulation pathways of natural killer (NK) cells and activated T cells [16]. Furthermore, CD226 is constitutively expressed on NK cells, CD4+ and CD8+ T cells, monocytes, platelets and certain B cells playing a pleiotropic role in the immune system [16,17], thus subtle changes in CD226 expression could be involved in SSc immune imbalance.

*CD226 *gene polymorphisms have been correlated with an increasing number of autoimmune pathologies. Thus, the *CD226 *rs763361/Gly307Ser non-synonymous polymorphism was first correlated to type 1 diabetes susceptibility [18,19], later to multiple autoimmune diseases [19,20] and recently, to SSc [21]. Interestingly, the minor allele rs763361*T encodes a non-synonymous mutation (Gly307Ser) in the cytoplasmic tail of CD226 protein (exon 7). In addition, the rs763361 glycine to serine substitution could interfere in the phosphorylation of CD226 at 322Tyr and 329Ser residues, and the downstream signaling pathway may be modulated by these posttranslational modifications [16,22].

In a recent study performed in SLE, a three-variant haplotype in *CD226 *gene, comprising *CD226 *rs763361-rs34794968-rs727088, was found to be associated with SLE and the authors proposed that rs727088 may be the single nucleotide polymorphism (SNP) with a functional influence on *CD226 *transcription levels [23].

The aim of this study was to test in a large European population the previously reported association of *CD226 *gene rs763361/Gly307Ser with SSc, and to analyze, for the first time, the role of two additional polymorphisms, rs34794968 and rs727088, and the effect of *CD226 *rs763361-rs34794968-rs727088 haplotype in SSc.

## Materials and methods

### Subjects

A total of 2,131 SSc cases and 3,966 controls from seven European Caucasian cohorts (Spain, Germany, the Netherlands, Italy, Sweden, the United Kingdom and Norway) were included in this study. Patients were diagnosed as having SSc using the criteria proposed by the 1980 ACR and/or LeRoy and Medsger criteria [24,25]. In addition, patients were classified as having limited or diffuse SSc as defined by LeRoy *et al. *[26]. The following clinical data was collected for ascertainment of clinical phenotype of all the patients with SSc: age, gender, disease duration and presence of SSc-associated autoantibodies, ATA and ACA. Pulmonary fibrosis was diagnosed by High Resolution Computed Tomography (HRCT). Considering the previously reported subphenotype associations, the subtype, autoantibody status and pulmonary fibrosis data were available for all the patients included in this report (Table S1 in Additional file 1 shows these clinical data). The control population consisted of unrelated healthy individuals recruited in the same geographical regions as SSc patients and matched by age, sex and ethnicity with the SSc patients groups. Local ethical committees from all the participating centers approved the study. Both patients and controls were included in the study after written informed consent.

### CD226 genotyping and statistical analysis

Three SNPs, rs763361 and rs3479968 located in exon 7, and rs727088 in the 3'UTR region were selected as genetic markers. The SNPs were analyzed by Taqman SNP genotyping assays in a 7900HT real-time polymerase chain reaction (PCR) System following the manufacturer's suggestions (Applied Biosystems, Foster City, CA, USA).

All cohorts were in Hardy-Weinberg equilibrium (HWE) at significance level = 0.01 for all the included SNPs. PLINK (v1.07) software [27] was used for individual population association tests (significance was calculated by 2 × 2 contingency tables and Fisher's exact test or χ^2 ^when necessary) and pooled analysis. Bonferroni and Benjamini and Hochberg false discovery rate method correction (FDR) were applied for multiple test correction [28]. In addition, the Breslow-Day test (BD test) was performed as implemented in PLINK to assess the homogeneity of the association among populations. Haplotype pooled analysis was performed by Haploview (Cambridge, MA, USA) and Statsdirect (Altrincham, Cheshire, UK) software. Power was calculated using the software Power Calculator for Genetic Studies 2006 and assuming an additive model [29].

## Results

### CD226 rs763361/Gly307Ser, rs34794968 and rs727088 analysis

Table 1 shows the genotype and allelic frequencies and pooled analyses of the three *CD226 *SNPs included in this report in the global disease and the considered subgroups. Tables S2 to S4 in Additional file 1 show the genotype and allele distribution of each of the tested variants in the seven analyzed European cohorts. The BD test revealed no statistically significant differences between the seven cohorts included, hence we performed a pooled analysis using the Mantel-Haenszel test under fixed effects for each of the tested polymorphisms. As opposed to Dieudé *et al. *[21], the pooled analysis of rs763361 showed no evidence of association with the whole set of SSc patients. Then, we interrogated the major SSc subphenotypes as defined by LeRoy *et al. *[26], and in addition by autoantibody status and by lung fibrosis as described in Dieudé *et al.*[21]. Allele frequencies in each subgroup were compared to control frequencies and no evidence of association was found at any of the considered subgroups. In addition, we observed no significant association in the pooled analyses of the *CD226 *rs34794968 and rs727088 genetic variants with neither the global disease nor with the considered phenotypic subgroups.

**Table 1:** Genotype and allele distribution of *CD226 *rs763361 (chr:18, 65,682,622 bp), rs34794968 (chr:18; 65,682,006 bp), rs727088 (chr:18, 65,681,419 bp) genetic variants and pooled analysis.

		Genotype, N (%)				Allele test			
				
SNP	Subgroup (N)	1/1	1/2	2/2	MAF (%)	*P*_MH_	*P*_FDR_BH_	OR [CI 95%]	*P*_BD_
rs763361	Controls (n = 3811)	906 (23.77)	1841 (48.31)	1064 (27.92)	47.93				
	SSc (n = 2023)	480 (23.73)	990 (48.94)	553 (27.34)	48.2	0.56	0.73	1.02 [0.95-1.10]	0.56
	lcSSc (n = 1397)	332 (23.77)	681 (48.75)	384 (27.49)	48.14	0.64	0.95	1.02 [0.94-1.11]	0.8
	dcSSc (n = 626)	148 (23.64)	309 (49.36)	169 (27.00)	48.32	0.6	0.94	1.03 [0.92-1.17]	0.14
	ACA+ (n = 797)	176 (22.08)	396 (49.69)	225 (28.23)	46.93	0.68	0.68	0.98 [0.88-1.09]	0.85
	ATA+ (n = 503)	133 (26.44)	239 (47.51)	131 (26.04)	50.2	0.22	0.28	1.09 [0.95-1.24]	0.63
	Fib+ (n = 729)	176 (24.14)	359 (49.25)	194 (26.61)	48.77	0.48	0.58	1.04 [0.93-1.17]	0.5

rs34794968	Controls (n = 3858)	669 (17.34)	1842 (47.74)	1347 (34.91)	41.21				
	SSc (n = 2060)	348 (16.89)	978 (47.48)	734 (35.63)	40.63	0.73	0.73	0.99 [0.91-1.07]	0.37
	lcSSc (n = 1422)	234 (16.46)	685 (48.17)	503 (35.37)	40.54	0.74	0.95	0.99 [0.90-1.08]	0.71
	dcSSc (n = 638)	114 (17.87)	293 (45.92)	231 (36.21)	40.83	0.94	0.94	1.00 [0.88-1.12]	0.05
	ACA+ (n = 816)	129 (15.81)	390 (47.79)	297 (36.40)	39.71	0.35	0.68	0.95 [0.85-1.06]	0.6
	ATA+ (n = 518)	100 (19.31)	249 (48.07)	169 (32.63)	43.34	0.28	0.28	1.08 [0.94-1.23]	0.38
	Fib+ (n = 755)	122 (16.16)	362 (47.95)	271 (35.89)	40.13	0.58	0.58	0.97 [0.87-1.09]	0.44

rs727088	Controls (n = 3815)	917 (24.04)	1869 (48.99)	1029 (26.97)	48.53				
	SSc (n = 2042)	489 (23.95)	1014 (49.66)	539 (26.40)	48.78	0.59	0.73	1.02 [0.95-1.10]	0.86
	lcSSc (n = 1409)	336 (23.85)	702 (49.82)	371 (26.33)	48.76	0.63	0.95	1.02 [0.94-1.11]	0.66
	dcSSc (n = 633)	153 (24.17)	312 (49.29)	168 (26.54)	48.82	0.71	0.94	1.02 [0.91-1.15]	0.26
	ACA+ (n = 814)	178 (21.87)	414 (50.86)	222 (27.27)	47.3	0.54	0.68	0.97 [0.87-1.08]	0.56
	ATA+ (n = 514)	138 (26.85)	250 (48.64)	126 (24.51)	51.17	0.14	0.28	1.10 [0.97-1.26]	0.61
	Fib+ (n = 739)	185 (25.03)	363 (49.12)	191 (25.85)	49.59	0.4	0.58	1.05 [0.94-1.17]	0.86

Controls are used as reference for all comparisons. ACA, anti-centromere antibodies; ATA, anti-topoisomerase antibodies; 95% CI, 95% confidence interval; dcSSc, diffuse cutaneous systemic sclerosis; Fib+, lung fibrosis positive SSc patients; lcSSc, limited cutaneous systemic sclerosis; MAF, minor allele frequency; NS, not significant; OR, odds ratio; *P*_BD_, Breslow-Day homogeneity test *p*-value; *P *_FDR_BH_, corrected *P-*value using Benjamini and Hochberg false discovery rate; *P*_MH_, allelic Mantel-Haenszel fixed effects model *P*-value; SSc, systemic sclerosis.

**Table 2:** Association of *CD226 *haplotype block in the Fib+ subset of systemic sclerosis patients.

rs763361	rs34794968	rs727088	Subgroup	MAF (%)	*P*_MH_	*P*_Bonf_	OR [CI 95%]	*P*_BD_
			Fib+	0.40				
T	A	G	CTRL	0.41	0.59	NS	0.97 [0.86-1.08]	0.45
			Fib-	0.41	0.76	NS	0.98 [0.86-1.11]	0.77

			Fib+	0.10				
T	C	G	CTRL	0.08	**1.59E-02**	**3.18E-02**	1.27 [1.05-1.54]	0.48
			Fib-	0.08	0.069	NS	1.24 [0.99-1.56]	0.51

			Fib+	0.50				
C	C	A	CTRL	0.51	0.44	NS	0.96 [0.86-1.07]	0.71
			Fib-	0.51	0.50	NS	0.95 [0.84-1.09]	0.44

For the haplotype-specific analyses, the odds ratio (OR) with 95% confidence interval (95% CI) was determined for each allele variant in the haplotype tested against all of the others pooled together using the Mantel-Haenszel test under fixed effects model, considering no single reference haplotype. CTRL, healthy controls; Fib-, lung fibrosis negative SSc patients; Fib+, lung fibrosis positive SSc patients; MAF, minor allele frequency; *P*_Bonf_, corrected *P*-value using Bonferroni multiple test correction.

### CD226 haplotype block analysis

Considering the *CD226 *haplotype block association described in SLE [23], we analyzed the possible effect of this haplotype block in SSc patients. In this analysis, considering only haplotypes with frequency > 5% in the pooled analysis, we reported no significant association with SSc susceptibility. However, stratification revealed that the TCG haplotype (SNP order: rs763361- rs34794968- rs727088) was over-represented in the lung fibrosis positive subgroup of patients. This haplotype, composed of the minor alleles of rs763361 and rs727088 polymorphisms and the major allele of rs34794968, was not equivalent to that associated with SLE susceptibility, but showed a modest risk effect in the lung fibrosis positive group of patients. This association with lung fibrosis remained significant even after performing Bonferroni multiple test correction, (*P*_Bonf _= 3.18E-02 OR 1.27 (1.05 to1.54), Table 2). Remarkably, a trend of association for the TCG haplotype was observed in the comparison between the lung fibrosis positive subset of patients and the fibrosis negative group of patients (Table 2). We consider that the lack of statistical significance may be possibly due to a reduced statistical power, as the lung fibrosis negative subgroup (*n *= 1,572) is smaller than the control group (*n *= 3,966).

## Discussion

We carried out a well-powered case-control study aiming to test the contribution of three *CD226 *genetic variants (rs763361, rs34794968 and rs727088) to SSc susceptibility and clinical phenotypes. We report the association of a *CD226 *three-variant haplotype with SSc-related pulmonary fibrosis. However, we observed a lack of individual association of these three *CD226 *polymorphisms with SSc or with its serological and clinical manifestations.

The allele and genotype frequencies observed in our control group were similar to those described in the HapMap public database phase 3 (CEPH: Utah residents with ancestry from northern and western Europe; abbreviation: CEU), and considerably comparable with those in Dieudé *et al. *report [21]. Although the present study did not confirm the previously reported association of rs763361 with SSc, we found that one of the haplotypes containing the rs763361*T allele showed evidence of association with the lung fibrosis positive group of patients [21]. We consider that the lack of association for rs763361 individually could have arisen due to a type II error (false negative), because our sample size is similar to the study by Dieudé *et al. *and reaches 99% power to detect the previously reported effect of the polymorphism. It is worth mentioning that Dieudé *et al. *analyzed this polymorphism both in an Italian and German replication cohorts, the latest being the most associated with the FA+ group. Consequently our FA+ German cohort, in spite of being smaller than the one in Dieudé *et al.*, showed the highest trend of association observed in this group (Table S1 in Additional file 1). Given the high power of our pooled analysis to detect similar associations to those reported in the previous SSc study, our data suggest that the previously reported effect for the association between *CD226 *rs763361*T allele and SSc susceptibility may be influenced by ethnical factors. Remarkably, Dieudé *et al. *studied the implication of the amino acid change encoded by rs763361*T in CD226 expression, but no significant differences were observed [21]. Our data together with SLE reports [23], suggest that haplotypic allele combinations might be considered for further functional studies.

Tao *et al. *described an increased susceptibility of NKT cells to apoptosis via CD95-CD95L or TCR-CD3 in NKT cells isolated from active SLE patients, and linked it with a deficient expression of CD226 [30]. Later, genetic studies revealed that rs727088 minor allele was part of a haplotype associated with SLE susceptibility and was responsible for a decrease in *CD226 *gene expression [23]. Despite experimental evidence of the role of rs727088 in *CD226 *transcription modulation in SLE, we detected no association signal of rs727088 or the SLE-risk haplotype with SSc susceptibility. These findings suggest that although *CD226 *is a common risk factor for SLE and SSc, the genetic variants in this gene may have potentially divergent roles in both diseases or the causal variant might be disease specific. Noteworthy, it is well-established that SSc and SLE share a number of associated loci, but the associated variants are not necessarily the same, for example in the human leukocyte antigen (HLA) region [31]. Considering that fibrosis is a main hallmark of SSc, the specific association of *CD226 *with SSc fibrosis-positive patients might reflect the influence of this locus in diverse pathways. Interestingly, *NLRP1 *a common autoimmune disease risk factor has been reported to be associated with the ATA and lung fibrosis-positive subgroups of SSc patients [32]. Moreover, *NLRP1 *was reported to contribute to SLE in families suffering SLE and vitiligo or other autoimmune or autoinflammatory diseases [33] and it has been recently found to be associated with SLE in a Brazilian population [34]. Nevertheless, rs2670660 and an rs12150220-rs2670660 haplotype were the associated variants in SLE, but only rs8182352 was associated with SSc-related pulmonary fibrosis (although both rs12150220 and rs2670660 were analyzed) [32,34]. Hence, although the population size in the SLE study is limited, this differential association in SLE of a novel SSc fibrosis risk factor is analogous to the reported *CD226 *results.

It is well-established that SSc patients exhibit reduced numbers and impaired function of NKT cells [35-37] and the highest CD226 expression levels in healthy donors are found in NKT cells [21,23]. Thus, the impact of the associated *CD226 *haplotype reported in this study on the functions of SSc patients' NK and NKT cells should be further explored. Furthermore, new therapeutic approaches based on anti-CD226 mAb treatment have already been tested in autoimmunity animal models [38], and CD226 has been recently implied in novel T cell activation pathways [39] and NK-driven tissue injury in SLE patients [40]. Hence, the implication of CD226 in cell-mediated cytotoxicity should be considered. In addition, the possibility of lung fibrosis being a marker of generalized internal organ damage, which was not analyzed in this study and represented a limitation of our findings, should be interrogated.

To date just a few loci have been reported to be associated with SSc-related pulmonary involvement, such as *IRF5 *[6], *STAT4 *[6], *TNFAIP3 *[41], *KCNA5 *[42], *NLRP1 *[32] and *HGF *[43]. Hence, we consider that the confirmation of *CD226 *as a pulmonary involvement marker might be valuable in the deciphering of the mechanisms that underlie the lung fibrosis process in SSc patients.

## Conclusions

Our data suggest that previously autoimmune-associated *CD226 *gene polymorphisms play a role in the SSc pulmonary fibrosis events in European Caucasian populations, and confirm *CD226 *as an important shared autoimmune factor in SSc.

## Abbreviations

ACA: anti-centromere autoantibodies; ATA: anti-topoisomerase antibodies; BD test: Breslow-Day test; CD226: cluster of differentiation 226; CEPH/CEU: Utah residents with ancestry from northern and western Europe; dcSSc: diffuse cutaneous systemic sclerosis; DNAM-1: DNAX accessory molecule 1; FDR: false discovery rate method correction; HLA: human leukocyte antigen; HRCT: high resolution computed tomography; HWE: Hardy-Weinberg equilibrium; lcSSc: limited cutaneous systemic sclerosis; PCR: polymerase chain reaction; PTA1: platelet and T-cell activation antigen 1; RA: rheumatoid arthritis; SLE: systemic lupus erythematosus; SNP: single nucleotide polymorphism; SSc: systemic sclerosis.

## Competing interests

The authors declare that they have no competing interests.

## Authors' contributions

LBC has contributed to the analysis and interpretation of data and to the drafting the manuscript. CPS, LB and JCB have participated in the acquisition of data and the drafting of the manuscript. CF, TRDJR and JM contributed to the conception and design of the study and have critically revised the manuscript. MCV, RRF, GE, PC, MTC, MJC, MAGG, EB, MCF, JN, CT, TW, AK, AJS, AMHV, RH, CL, JMvL, MMC, AH, CPD, BPK have been involved in the acquisition of data and the revision of the manuscript. All authors read and approved the final manuscript.

## Supplementary Material

Additional file 1 **Population specific characteristics and genotype and allelic distribution of the three analyzed variants in each population**. This file contains: Table S1 showing the population specific composition of the complete SSc set of patients for the analyzed features of the disease; Tables S2 to 4 showing the genotype and allele distributions of *CD226 *rs763361, rs34794968 and rs727088 genetic variants in seven European cohorts. Click here for file
