# Supplementary material for: A multicenter study confirms CD226 gene association with systemic sclerosis-related pulmonary fibrosis
Source: Arthritis Res Ther. 2012 Apr 24;14(2):R85. doi: 10.1186/ar3809 (PMC3446459; doi:10.1186/ar3809)
Supplement: Additional file 1 — Population specific characteristics and genotype and allelic distribution of the three analyzed variants in each population. This file contains: Table S1 showing the population specific composition of the complete SSc set of patients for the analyzed features of the disease; Tables S2 to 4 showing the genotype and allele distributions of CD226 rs763361, rs34794968 and rs727088 genetic variants in seven European cohorts. [file ar3809-S1.DOC]

**Table s1**. Population specific composition of the complete SSc set of patients for the analyzed features of the disease.

| **Population** | **lcSSc %** | **dcSSc %** | **ACA+ %** | **ATA+ %** | **Fib +%** |
| --- | --- | --- | --- | --- | --- |
| Spain | 67.95 | 32.05 | 45.54 | 22.82 | 25.25 |
| Germany | 57.14 | 42.86 | 40.00 | 30.20 | 34.90 |
| The Netherlands | 69.70 | 30.30 | 22.90 | 28.96 | 47.47 |
| Italy | 74.33 | 25.67 | 45.50 | 34.50 | 33.67 |
| Sweden | 76.70 | 23.30 | 27.84 | 17.05 | 52.27 |
| The United Kingdom | 69.27 | 30.73 | 36.07 | 19.79 | 39.32 |
| Norway | 65.45 | 34.55 | 53.64 | 14.55 | 39.09 |
| **Overall** | **68.34** | **31.66** | **40.01** | **25.21** | **35.02** |

lcSSc: limited cutaneous systemic sclerosis; dcSSc: diffuse cutaneous systemic sclerosis; ACA+: anti-centromere autoantibody positive patients; ATA+: anti-topoisomerase autoantibody positive patients. NA: not available data.

**Table s2**. Genotype and allele distribution of *CD226* rs763361 (chr:18, 65,682,622bp) genetic variant in seven European cohorts.

|  |  | **Genotype, N (%)** | | |  | **Allele test** | |
| --- | --- | --- | --- | --- | --- | --- | --- |
| **CHR** | **Subgroup (N)** | **1/1** | **1/2** | **2/2** | **MAF (%)** | ***P*-value** | **OR [CI 95%]** |
| Spain | Controls (n=1342) | 298 (22.21) | 640 (47.69) | 404 (30.10) | 46.05 |  |  |
|  | SSc (n=793) | 181 (22.82) | 380 (47.92) | 232 (29.26) | 46.78 | 0.64 | 1.03 [0.91-1.17] |
|  | lcSSc (n=536) | 125 (23.32) | 262 (48.88) | 149 (27.80) | 47.76 | 0.34 | 1.07 [0.93-1.23] |
|  | dcSSc (n=257) | 56 (21.79) | 118 (45.91) | 83 (32.30) | 44.75 | 0.59 | 0.95 [0.78-1.15] |
|  | ACA+ (n=365) | 81 (22.19) | 183 (50.14) | 101 (27.67) | 47.26 | 0.56 | 1.05 [0.89-1.24] |
|  | ATA+ (n=176) | 40 (22.73) | 80 (45.45) | 56 (31.82) | 45.45 | 0.83 | 0.98 [0.78-1.22] |
|  | Fib+ (n=206) | 47 (22.82) | 100 (48.54) | 59 (28.64) | 47.09 | 0.69 | 1.04 [0.85-1.28] |
| Germany | Controls (n=271) | 60 (22.14) | 141 (52.03) | 70 (25.83) | 48.15 |  |  |
|  | SSc (n=134) | 32 (23.88) | 53 (39.55) | 49 (36.57) | 43.66 | 0.23 | 0.83 [0.62-1.12] |
|  | lcSSc (n=73) | 19 (26.03) | 26 (35.62) | 28 (38.36) | 43.84 | 0.35 | 0.84 [0.58-1.21] |
|  | dcSSc (n=61) | 13 (21.31) | 27 (44.26) | 21 (34.43) | 43.44 | 0.35 | 0.83 [0.56-1.23] |
|  | ACA+ (n=42) | 10 (23.81) | 19 (45.24) | 13 (30.95) | 46.43 | 0.77 | 0.93 [0.59-1.48] |
|  | ATA+ (n=37) | 9 (24.32) | 17 (45.95) | 11 (29.73) | 47.3 | 0.89 | 0.97 [0.59-1.57] |
|  | Fib+ (n=65) | 14 (21.54) | 24 (36.92) | 27 (41.54) | 40 | 0.09 | 0.72 [0.49-1.06] |
| The Netherlands | Controls (n=538) | 127 (23.61) | 269 (50.00) | 142 (26.39) | 48.61 |  |  |
|  | SSc (n=272) | 56 (20.59) | 155 (56.99) | 61 (22.43) | 49.08 | 0.86 | 1.02 [0.83-1.25] |
|  | lcSSc (n=190) | 39 (20.53) | 107 (56.32) | 44 (23.16) | 48.68 | 0.98 | 1.00 [0.79-1.27] |
|  | dcSSc (n=82) | 17 (20.73) | 48 (58.54) | 17 (20.73) | 50 | 0.74 | 1.06 [0.76-1.47] |
|  | ACA+ (n=60) | 12 (20.00) | 33 (55.00) | 15 (25.00) | 47.5 | 0.82 | 0.96 [0.66-1.40] |
|  | ATA+ (n=79) | 18 (22.78) | 48 (60.76) | 13 (16.46) | 53.16 | 0.28 | 1.20 [0.86-1.68] |
|  | Fib+ (n=127) | 24 (18.90) | 76 (59.84) | 27 (21.26) | 48.82 | 0.95 | 1.01 [0.77-1.33] |
| Italy | Controls (n=777) | 201 (25.87) | 356 (45.82) | 220 (28.31) | 48.78 |  |  |
|  | SSc (n=306) | 66 (21.57) | 156 (50.98) | 84 (27.45) | 47.06 | 0.47 | 0.93 [0.77-1.13] |
|  | lcSSc (n=223) | 49 (21.97) | 112 (50.22) | 62 (27.80) | 47.09 | 0.53 | 0.93 [0.76-1.15] |
|  | dcSSc (n=83) | 17 (20.48) | 44 (53.01) | 22 (26.51) | 46.99 | 0.66 | 0.93 [0.68-1.28] |
|  | ACA+ (n=132) | 28 (21.21) | 71 (53.79) | 33 (25.00) | 48.11 | 0.84 | 0.97 [0.75-1.26] |
|  | ATA+ (n=133) | 29 (21.80) | 61 (45.86) | 43 (32.33) | 44.74 | 0.22 | 0.85 [0.65-1.10] |
|  | Fib+ (n=108) | 25 (23.15) | 52 (48.15) | 31 (28.70) | 47.22 | 0.67 | 0.94 [0.71-1.25] |
| Sweden | Controls (n=265) | 57 (21.51) | 137 (51.70) | 71 (26.79) | 47.36 |  |  |
|  | SSc (n=155) | 32 (20.65) | 83 (53.55) | 40 (25.81) | 47.42 | 0.99 | 1.00 [0.76-1.33] |
|  | lcSSc (n=117) | 26 (22.22) | 59 (50.43) | 32 (27.35) | 47.44 | 0.98 | 1.00 [0.74-1.37] |
|  | dcSSc (n=38) | 6 (15.79) | 24 (63.16) | 8 (21.05) | 47.37 | 1.00 | 1.00 [0.62-1.62] |
|  | ACA+ (n=42) | 10 (23.81) | 17 (40.48) | 15 (35.71) | 44.05 | 0.57 | 0.88 [0.55-1.39] |
|  | ATA+ (n=24) | 3 (12.50) | 14 (58.33) | 7 (29.17) | 41.67 | 0.45 | 0.79 [0.44-1.45] |
|  | Fib+ (n=81) | 18 (22.22) | 43 (53.09) | 20 (24.69) | 48.77 | 0.75 | 1.06 [0.74-1.51] |
| The United Kingdom | Controls (n=366) | 76 (20.77) | 184 (50.27) | 106 (28.96) | 45.9 |  |  |
|  | SSc (n=270) | 72 (26.67) | 125 (46.30) | 73 (27.04) | 49.81 | 0.17 | 1.17 [0.94-1.46] |
|  | lcSSc (n=196) | 47 (23.98) | 88 (44.90) | 61 (31.12) | 46.43 | 0.87 | 1.02 [0.80-1.31] |
|  | dcSSc (n=74) | 25 (33.78) | 37 (50.00) | 12 (16.22) | 58.78 | **4.23E-03** | 1.68 [1.18-2.41] |
|  | ACA+ (n=105) | 18 (17.14) | 51 (48.57) | 36 (34.29) | 41.43 | 0.25 | 0.83 [0.61-1.14] |
|  | ATA+ (n=42) | 15 (35.71) | 16 (38.10) | 11 (26.19) | 54.76 | 0.12 | 1.43 [0.91-2.25] |
|  | Fib+ (n=103) | 27 (26.21) | 50 (48.54) | 26 (25.24) | 50.49 | 0.24 | 1.20 [0.88-1.64] |
| Norway | Controls (n=252) | 68 (26.98) | 114 (45.24) | 70 (27.78) | 49.6 |  |  |
|  | SSc (n=93) | 23 (24.73) | 38 (40.86) | 32 (34.41) | 45.16 | 0.30 | 0.84 [0.60-1.17] |
|  | lcSSc (n=62) | 14 (22.58) | 27 (43.55) | 21 (33.87) | 44.35 | 0.29 | 0.81 [0.55-1.20] |
|  | dcSSc (n=31) | 9 (29.03) | 11 (35.48) | 11 (35.48) | 46.77 | 0.67 | 0.89 [0.53-1.52] |
|  | ACA+ (n=51) | 12 (23.53) | 22 (43.14) | 17 (33.33) | 45.1 | 0.41 | 0.83 [0.54-1.28] |
|  | ATA+ (n=12) | 5 (41.67) | 3 (25.00) | 4 (33.33) | 54.17 | 0.66 | 1.20 [0.53-2.73] |
|  | Fib+ (n=39) | 10 (25.64) | 14 (35.90) | 15 (38.46) | 43.59 | 0.26 | 0.76 [0.47-1.23] |

Controls are used as reference for all comparisons. MAF: minor allele frequency; *P*-value: allelic Chisq uncorrected *p*-value; SSc: systemic sclerosis; dcSSc: diffuse cutaneous systemic sclerosis; ATA: anti-topoisomerase antibodies; Fib+: lung fibrosis positive SSc patients.

**Table s3**. Genotype and allele distribution of *CD226* rs34794968 (chr:18; 65,682,006bp) genetic variant in seven European cohorts.

|  |  | **Genotype, N (%)** |  | **Allele test** |  | | |
| --- | --- | --- | --- | --- | --- | --- | --- |
| **CHR** | **Subgroup (N)** | **1/1** | **1/2** | **2/2** | **MAF (%)** | ***P*-value** | **OR [CI 95%]** |
| Spain | Controls (n=1377) | 230 (16.70) | 644 (46.77) | 503 (36.53) | 40.09 |  |  |
|  | SSc (n=786) | 138 (17.56) | 356 (45.29) | 292 (37.15) | 40.2 | 0.94 | 1.01 [0.89-1.14] |
|  | lcSSc (n=531) | 96 (18.08) | 244 (45.95) | 191 (35.97) | 41.05 | 0.59 | 1.04 [0.90-1.20] |
|  | dcSSc (n=255) | 42 (16.47) | 112 (43.92) | 101 (39.61) | 38.43 | 0.48 | 0.93 [0.77-1.13] |
|  | ACA+ (n=363) | 65 (17.91) | 168 (46.28) | 130 (35.81) | 41.05 | 0.64 | 1.04 [0.88-1.23] |
|  | ATA+ (n=175) | 28 (16.00) | 80 (45.71) | 67 (38.29) | 38.86 | 0.66 | 0.95 [0.76-1.19] |
|  | Fib+ (n=205) | 34 (16.59) | 97 (47.32) | 74 (36.10) | 40.24 | 0.95 | 1.01 [0.81-1.24] |
| Germany | Controls (n=277) | 41 (14.80) | 150 (54.15) | 86 (31.05) | 41.88 |  |  |
|  | SSc (n=154) | 23 (14.94) | 68 (44.16) | 63 (40.91) | 37.01 | 0.16 | 0.82 [0.61-1.09] |
|  | lcSSc (n=82) | 12 (14.63) | 38 (46.34) | 32 (39.02) | 37.8 | 0.35 | 0.84 [0.59-1.21] |
|  | dcSSc (n=72) | 11 (15.28) | 30 (41.67) | 31 (43.06) | 36.11 | 0.21 | 0.78 [0.54-1.15] |
|  | ACA+ (n=50) | 5 (10.00) | 28 (56.00) | 17 (34.00) | 38 | 0.47 | 0.85 [0.55-1.32] |
|  | ATA+ (n=42) | 8 (19.05) | 18 (42.86) | 16 (38.10) | 40.48 | 0.81 | 0.94 [0.59-1.51] |
|  | Fib+ (n=80) | 10 (12.50) | 33 (41.25) | 37 (46.25) | 33.12 | **0.046** | 0.69 [0.47-1.00] |
| The Netherlands | Controls (n=528) | 100 (18.94) | 251 (47.54) | 177 (33.52) | 42.71 |  |  |
|  | SSc (n=273) | 38 (13.92) | 138 (50.55) | 97 (35.53) | 39.19 | 0.18 | 0.86 [0.70-1.07] |
|  | lcSSc (n=191) | 24 (12.57) | 102 (53.40) | 65 (34.03) | 39.27 | 0.24 | 0.87 [0.68-1.10] |
|  | dcSSc (n=82) | 14 (17.07) | 36 (43.90) | 32 (39.02) | 39.02 | 0.37 | 0.86 [0.61-1.20] |
|  | ACA+ (n=62) | 6 (9.68) | 30 (48.39) | 26 (41.94) | 33.87 | 0.06 | 0.69 [0.46-1.02] |
|  | ATA+ (n=82) | 14 (17.07) | 39 (47.56) | 29 (35.37) | 40.85 | 0.65 | 0.93 [0.66-1.29] |
|  | Fib+ (n=131) | 14 (10.69) | 73 (55.73) | 44 (33.59) | 38.55 | 0.22 | 0.84 [0.64-1.11] |
| Italy | Controls (n=782) | 162 (20.72) | 371 (47.44) | 249 (31.84) | 44.44 |  |  |
|  | SSc (n=320) | 66 (20.63) | 163 (50.94) | 91 (28.44) | 46.09 | 0.48 | 1.07 [0.89-1.29] |
|  | lcSSc (n=234) | 50 (21.37) | 115 (49.15) | 69 (29.49) | 45.94 | 0.57 | 1.06 [0.86-1.31] |
|  | dcSSc (n=86) | 16 (18.60) | 48 (55.81) | 22 (25.58) | 46.51 | 0.60 | 1.09 [0.79-1.49] |
|  | ACA+ (n=139) | 27 (19.42) | 69 (49.64) | 43 (30.94) | 44.24 | 0.95 | 0.99 [0.77-1.28] |
|  | ATA+ (n=136) | 34 (25.00) | 71 (52.21) | 31 (22.79) | 51.1 | **0.042** | 1.31 [1.01-1.69] |
|  | Fib+ (n=116) | 27 (23.28) | 54 (46.55) | 35 (30.17) | 46.55 | 0.55 | 1.09 [0.83-1.44] |
| Sweden | Controls (n=271) | 33 (12.18) | 136 (50.18) | 102 (37.64) | 37.27 |  |  |
|  | SSc (n=159) | 20 (12.58) | 81 (50.94) | 58 (36.48) | 38.05 | 0.82 | 1.03 [0.78-1.38] |
|  | lcSSc (n=122) | 15 (12.30) | 63 (51.64) | 44 (36.07) | 38.11 | 0.82 | 1.04 [0.76-1.42] |
|  | dcSSc (n=37) | 5 (13.51) | 18 (48.65) | 14 (37.84) | 37.84 | 0.92 | 1.03 [0.62-1.69] |
|  | ACA+ (n=45) | 5 (11.11) | 21 (46.67) | 19 (42.22) | 34.44 | 0.61 | 0.88 [0.55-1.41] |
|  | ATA+ (n=27) | 2 (7.41) | 16 (59.26) | 9 (33.33) | 37.04 | 0.97 | 0.99 [0.55-1.77] |
|  | Fib+ (n=81) | 12 (14.81) | 39 (48.15) | 30 (37.04) | 38.89 | 0.71 | 1.07 [0.75-1.54] |
| The United Kingdom | Controls (n=362) | 55 (15.19) | 170 (46.96) | 137 (37.85) | 38.67 |  |  |
|  | SSc (n=271) | 47 (17.34) | 132 (48.71) | 92 (33.95) | 41.7 | 0.28 | 1.13 [0.90-1.42] |
|  | lcSSc (n=197) | 28 (14.21) | 93 (47.21) | 76 (38.58) | 37.82 | 0.78 | 0.96 [0.75-1.24] |
|  | dcSSc (n=74) | 19 (25.68) | 39 (52.70) | 16 (21.62) | 52.03 | **2.61E-03** | 1.72 [1.21-2.45] |
|  | ACA+ (n=105) | 13 (12.38) | 51 (48.57) | 41 (39.05) | 36.67 | 0.60 | 0.92 [0.67-1.26] |
|  | ATA+ (n=42) | 8 (19.05) | 22 (52.38) | 12 (28.57) | 45.24 | 0.24 | 1.31 [0.83-2.07] |
|  | Fib+ (n=103) | 16 (15.53) | 51 (49.51) | 36 (34.95) | 40.29 | 0.67 | 1.07 [0.78-1.47] |
| Norway | Controls (n=261) | 48 (18.39) | 120 (45.98) | 93 (35.63) | 41.38 |  |  |
|  | SSc (n=97) | 16 (16.49) | 40 (41.24) | 41 (42.27) | 37.11 | 0.30 | 0.84 [0.60-1.17] |
|  | lcSSc (n=65) | 9 (13.85) | 30 (46.15) | 26 (40.00) | 36.92 | 0.35 | 0.83 [0.56-1.23] |
|  | dcSSc (n=32) | 7 (21.88) | 10 (31.25) | 15 (46.88) | 37.5 | 0.55 | 0.85 [0.50-1.45] |
|  | ACA+ (n=52) | 8 (15.38) | 23 (44.23) | 21 (40.38) | 37.5 | 0.46 | 0.85 [0.55-1.31] |
|  | ATA+ (n=14) | 6 (42.86) | 3 (21.43) | 5 (35.71) | 53.57 | 0.20 | 1.64 [0.76-3.51] |
|  | Fib+ (n=39) | 9 (23.08) | 15 (38.46) | 15 (38.46) | 42.31 | 0.88 | 1.04 [0.64-1.68] |

Controls are used as reference for all comparisons. MAF: minor allele frequency; *P*-value: allelic Chisq uncorrected *p*-value; SSc: systemic sclerosis; dcSSc: diffuse cutaneous systemic sclerosis; ATA: anti-topoisomerase antibodies; Fib+: lung fibrosis positive SSc patients.

**Table s4**. Genotype and allele distribution of *CD226* rs727088 (chr:18, 65,681,419bp) genetic variant in seven European cohorts.

|  |  | **Genotype, N (%)** |  | **Allele test** |  | | |
| --- | --- | --- | --- | --- | --- | --- | --- |
| **CHR** | **Subgroup (N)** | **1/1** | **1/2** | **2/2** | **MAF (%)** | ***P*-value** | **OR [CI 95%]** |
| Spain | Controls (n=1356) | 301 (22.20) | 661 (48.75) | 394 (29.06) | 46.57 |  |  |
|  | SSc (n=784) | 181 (23.09) | 380 (48.47) | 223 (28.44) | 47.32 | 0.64 | 1.03 [0.91-1.17] |
|  | lcSSc (n=533) | 128 (24.02) | 263 (49.34) | 142 (26.64) | 48.69 | 0.24 | 1.09 [0.94-1.25] |
|  | dcSSc (n=251) | 53 (21.12) | 117 (21.12) | 81 (46.61) | 44.42 | 0.38 | 0.92 [0.76-1.11] |
|  | ACA+ (n=365) | 84 (23.01) | 184 (50.41) | 97 (26.58) | 48.22 | 0.43 | 1.07 [0.91-1.26] |
|  | ATA+ (n=178) | 41 (23.03) | 82 (46.07) | 55 (30.90) | 46.07 | 0.86 | 0.98 [0.79-1.22] |
|  | Fib+ (n=203) | 48 (23.65) | 96 (47.29) | 59 (29.06) | 47.29 | 0.79 | 1.03 [0.84-1.27] |
| Germany | Controls (n=270) | 63 (23.33) | 141 (52.22) | 66 (24.44) | 49.44 |  |  |
|  | SSc (n=149) | 36 (24.16) | 69 (46.31) | 44 (29.53) | 47.32 | 0.56 | 0.92 [0.69-1.22] |
|  | lcSSc (n=79) | 17 (21.52) | 36 (45.57) | 26 (32.91) | 44.3 | 0.26 | 0.81 [0.57-1.16] |
|  | dcSSc (n=70) | 19 (27.14) | 33 (27.14) | 18 (47.14) | 50.71 | 0.79 | 1.05 [0.73-1.53] |
|  | ACA+ (n=47) | 8 (17.02) | 26 (55.32) | 13 (27.66) | 44.68 | 0.39 | 0.83 [0.53-1.28] |
|  | ATA+ (n=42) | 9 (21.43) | 20 (47.62) | 13 (30.95) | 45.24 | 0.36 | 0.81 [0.51-1.28] |
|  | Fib+ (n=74) | 19 (25.68) | 32 (43.24) | 23 (31.08) | 47.3 | 0.64 | 0.92 [0.64-1.32] |
| The Netherlands | Controls (n=535) | 130 (24.30) | 270 (50.47) | 135 (25.23) | 49.53 |  |  |
|  | SSc (n=280) | 57 (20.36) | 159 (56.79) | 64 (22.86) | 48.75 | 0.76 | 0.97 [0.79-1.19] |
|  | lcSSc (n=195) | 39 (20.00) | 112 (57.44) | 44 (22.56) | 48.72 | 0.78 | 0.97 [0.77-1.22] |
|  | dcSSc (n=85) | 18 (21.18) | 47 (21.18) | 20 (55.29) | 48.82 | 0.86 | 0.97 [0.70-1.34] |
|  | ACA+ (n=63) | 12 (19.05) | 35 (55.56) | 16 (25.40) | 46.83 | 0.57 | 0.90 [0.62-1.30] |
|  | ATA+ (n=82) | 16 (19.51) | 51 (62.20) | 15 (18.29) | 50.61 | 0.80 | 1.04 [0.75-1.45] |
|  | Fib+ (n=133) | 26 (19.55) | 78 (58.65) | 29 (21.80) | 48.87 | 0.85 | 0.97 [0.74-1.27] |
| Italy | Controls (n=764) | 221 (28.93) | 350 (45.81) | 193 (25.26) | 51.83 |  |  |
|  | SSc (n=304) | 85 (27.96) | 157 (51.64) | 62 (20.39) | 53.78 | 0.42 | 1.08 [0.89-1.30] |
|  | lcSSc (n=220) | 64 (29.09) | 111 (50.45) | 45 (20.45) | 54.32 | 0.36 | 1.10 [0.89-1.37] |
|  | dcSSc (n=84) | 21 (54.76) | 46 (20.24) | 17 (20.24) | 52.38 | 0.89 | 1.02 [0.74-1.40] |
|  | ACA+ (n=134) | 35 (26.12) | 72 (53.73) | 27 (20.15) | 52.99 | 0.73 | 1.05 [0.80-1.36] |
|  | ATA+ (n=129) | 42 (32.56) | 61 (47.29) | 26 (20.16) | 56.2 | 0.19 | 1.19 [0.91-1.55] |
|  | Fib+ (n=109) | 34 (31.19) | 52 (47.71) | 23 (21.10) | 55.05 | 0.37 | 1.14 [0.85-1.51] |
| Sweden | Controls (n=263) | 53 (20.15) | 143 (54.37) | 67 (25.48) | 47.34 |  |  |
|  | SSc (n=155) | 32 (20.65) | 82 (52.90) | 41 (26.45) | 47.1 | 0.95 | 0.99 [0.75-1.31] |
|  | lcSSc (n=119) | 26 (21.85) | 61 (51.26) | 32 (26.89) | 47.48 | 0.97 | 1.01 [0.74-1.37] |
|  | dcSSc (n=36) | 6 (16.67) | 21 (16.67) | 9 (58.33) | 45.83 | 0.81 | 0.94 [0.57-1.54] |
|  | ACA+ (n=45) | 9 (20.00) | 19 (42.22) | 17 (37.78) | 41.11 | 0.27 | 0.78 [0.49-1.22] |
|  | ATA+ (n=27) | 4 (14.81) | 16 (59.26) | 7 (25.93) | 44.44 | 0.68 | 0.89 [0.51-1.56] |
|  | Fib+ (n=80) | 17 (21.25) | 41 (51.25) | 22 (27.50) | 46.88 | 0.92 | 0.98 [0.69-1.40] |
| The United Kingdom | Controls (n=366) | 80 (21.86) | 183 (50.00) | 103 (28.14) | 46.86 |  |  |
|  | SSc (n=273) | 72 (26.37) | 128 (46.89) | 73 (26.74) | 49.82 | 0.30 | 1.13 [0.90-1.41] |
|  | lcSSc (n=197) | 47 (23.86) | 89 (45.18) | 61 (30.96) | 46.45 | 0.90 | 0.98 [0.77-1.26] |
|  | dcSSc (n=76) | 25 (32.89) | 39 (32.89) | 12 (51.32) | 58.55 | 0.01 | 1.60 [1.13-2.28] |
|  | ACA+ (n=107) | 18 (16.82) | 53 (49.53) | 36 (33.64) | 41.59 | 0.17 | 0.81 [0.59-1.10] |
|  | ATA+ (n=43) | 15 (34.88) | 18 (41.86) | 10 (23.26) | 55.81 | 0.12 | 1.43 [0.91-2.25] |
|  | Fib+ (n=103) | 26 (25.24) | 52 (50.49) | 25 (24.27) | 50.49 | 0.36 | 1.16 [0.85-1.58] |
| Norway | Controls (n=261) | 69 (26.44) | 121 (46.36) | 71 (27.20) | 49.62 |  |  |
|  | SSc (n=97) | 26 (26.80) | 39 (40.21) | 32 (32.99) | 46.91 | 0.52 | 0.90 [0.64-1.25] |
|  | lcSSc (n=66) | 15 (22.73) | 30 (45.45) | 21 (31.82) | 45.45 | 0.39 | 0.85 [0.58-1.24] |
|  | dcSSc (n=31) | 11 (35.48) | 9 (35.48) | 11 (29.03) | 50 | 0.95 | 1.02 [0.60-1.72] |
|  | ACA+ (n=53) | 12 (22.64) | 25 (47.17) | 16 (30.19) | 46.23 | 0.52 | 0.87 [0.57-1.33] |
|  | ATA+ (n=13) | 4 (30.77) | 2 (15.38) | 7 (53.85) | 38.46 | 0.24 | 0.62 [0.27-1.38] |
|  | Fib+ (n=37) | 10 (27.03) | 12 (32.43) | 15 (40.54) | 43.24 | 0.25 | 0.75 [0.46-1.23] |

Controls are used as reference for all comparisons. MAF: minor allele frequency; *P*-value: allelic Chisq uncorrected *p*-value; SSc: systemic sclerosis; dcSSc: diffuse cutaneous systemic sclerosis; ATA: anti-topoisomerase antibodies; Fib+: lung fibrosis positive SSc patients.
